# Iron-Based Metal–Organic Frameworks for the Removal of Different Organic and Inorganic Arsenic Species from Water: Kinetic and Adsorption Studies

**DOI:** 10.3390/molecules30214198

**Published:** 2025-10-27

**Authors:** Afef Azri, Khaled Walha, Claudia Fontàs, José-Elias Conde-González, Eladia M. Peña-Méndez, Andreas Seubert, Victoria Salvadó

**Affiliations:** 1 Department de Química, Facultat de Ciències, Universitat de Girona, C/Mᵃ Aurèlia Capmany, 69, 17003 Girona, Spain; afef.azri@udl.cat (A.A.); claudia.fontas@udg.edu (C.F.); 2 Laboratory of Material Sciences and Environment, Faculty of Sciences, University of Sfax, Sfax 3029, Tunisia; khaled.walha@fss.usf.tn; 3 Departamento de Química, Unidad Departamental de Química Analítica, Facultad de Ciencias, Universidad de La Laguna, Avda. Astrofísico Fco. Sánchez, s/n, 38206 La Laguna, Spain; jconde@ull.edu.es (J.-E.C.-G.); empena@ull.edu.es (E.M.P.-M.); 4 Faculty of Chemistry, Analytical Chemistry, University of Marburg, Hans-Meerwein-Str. 4, 35043 Marburg, Germany; seubert@staff.uni-marburg.de

**Keywords:** organoarsenicals, arsenate, arsenite, iron-trimesate MOFs, adsorption isotherm, kinetics, reuse

## Abstract

Basolite^®^ F300 and synthetic nano-{Fe-BTC} MOFs, two iron-trimesate MOFs, have been investigated, demonstrating broad pH range adsorption for monomethylarsenate (MMA), cacodylic acid (DMAA), 4-aminophenylarsonate (ASA), and arsenate, while arsenite adsorption was notable at pH > 9.5. A similar uptake trend was found for both MOFs, with Basolite^®^ F300 being the more effective given its higher porosity and greater surface area. Pseudo-second-order kinetic models were followed by MMA, DMAA, ASA, and As(V), suggesting a chemisorption mechanism with arsenic species diffusion into MOF pores as the controlling step. Equilibrium data for DMAA and ASA fit the Langmuir model whereas MMA adsorption fits the Redlich–Peterson model. The uptake of MMA, DMAA, and ASA by both Fe-MOFs is mainly attributed to their coordination with Fe(III). Aromatic units in ASA enhance adsorption through П-П stacking interactions. The competition between all arsenic species for the sorption sites of the Fe-MOFs led to an uptake decrease of 10% for MMA and ASA and higher than 30% for DMAA and As(V) with respect to the individual uptakes. The Fe-MOFs can be reused for four cycles by washing with acidic methanol. Basolite^®^ F300 and synthetic nano-{Fe-BTC} effectively removed organic and inorganic arsenic species, exhibiting rapid adsorption, selective uptake, stability, and easy regeneration.

## 1. Introduction

Arsenic is a toxic metalloid which is present in the environment around the world due to both geological conditions and anthropogenic activity. Arsenic exposure is monitored and managed by regulatory agencies and public health organizations, such as the World Health Organization (WHO), which has established drinking water limits of 10 µg of arsenic per litre due to its condition as a first group carcinogen [1,2,3,4]. Arsenic compounds are mostly found in two forms: inorganic species (arsenite and arsenate As(V)) and organic forms (methylated and aromatic organoarsenicals), which exhibit different degrees of toxicity [5]. Inorganic arsenic species are toxic and arsenites are up to 60 times more toxic than arsenates. Organoarsenicals, which are considered non-toxic, can be transformed into harmful inorganic equivalents: As(III) and As(V) [6].

Organoarsenicals are widely used in agricultural and farming. Methylated arsenics, such as monomethylarsenic acid (MMA) and cacodylic acid (DMA), are used as herbicides and pesticides whereas aromatic 4-Aminobenzenearsonic acid (ASA) and roxarsone (ROX) are used as feed additives in the poultry and pork industries [7,8]. The biotransformation of water-soluble organoarsenical species into more harmful inorganic species is a major concern both for public health and the conservation of ecosystems [5,9,10]. It is therefore imperative to address arsenic contamination comprehensively with the aim of mitigating the associated risks in aquatic environments, focusing on all arsenic species given the metabolic and biodegradation changes that take place.

Available removal technologies for arsenic removal include chemical precipitation, ion exchange, membrane filtration, phytoremediation, electrocoagulation, and adsorption [11,12,13,14,15,16].

Among these, adsorption is recognized as one of the simplest and most cost-efficient technologies [14]. A recent review extensively discusses the use of various adsorbents for arsenic removal, including metal–organic frameworks (MOFs) [17]. MOFs are crystalline materials consisting of clusters of metal ions (Lewis acid) as well as organic ligands (Lewis base), which have a high adsorption capacity due to their porosity, a large specific surface area, tuneable pore size, and high thermal stability [18,19]. MOFs can be easily synthesized and contain coordinated unsaturated sites (CUSs) created by the empty Lewis acid sites on the metal [20]. The removal of aquatic arsenic species by different MOFs was systematically reviewed and summarized [21,22]. Of the different MOFs, Fe-based MOFs have been widely investigated for arsenic adsorption as they show better performance due to the high affinity of arsenic species for iron [23,24,25], the tuneable pore size, the environment of internal pores, and structural characteristics as well as the fact that iron is abundant in the earth’s crust, less toxic, and costs less than other transition metals. The most recent developments in the facile synthesis and potential application of Fe-based MOF adsorbents for the elimination of As ions have been reviewed [26].

The main Fe-based MOFs reported for arsenic removal are those of the Materials of Institute Lavoisier (MIL) series: MIL-100-Fe [27], MIL-53 [28], and MIL-101-Fe [6]. The framework of MIL-100 is made up of three octahedra sharing a μ3-O common vertex interconnected by 1,3,5-benzene tricarboxylate (BTC) ligands, while MIL-101 has a zeolite-like structure with 1,4-benzene dicarboxylate (1,4-BDC) combined with similar trimers [29].

Organoarsenicals such as ASA and ROX were found to be effectively adsorbed by MIL-100-Fe, surpassing the efficiency of conventional adsorbents such as activated carbon, zeolite Y, goethite, and other MOFs (MIL-101-Cr and MIL-53-Cr) across all adsorption times [27]. MIL-101(Fe) exhibited high adsorption capacities for arsenate, ROX, p-ASA, and DMA, which followed the order ROX > p-ASA > As(V) > DMA since the presence of aromatic units in ROX and p-ASA improved the sorption efficiency through hydrogen bonding and pi–pi interactions [6]. MIL-88A on cotton fibres have shown adsorption capacities for various arsenic pollutants with the order from high to low as follows: p-ASA > ROX > As(V) > As(III) [30]. Recently, thermal activation of MIL-88A was found to improve its adsorption capacity with maximum adsorption capacities following the order As(V) > p-ASA > ROX > DMA, which were determined by their different molecular sizes and adsorption mechanisms [30]. Most of these studies were performed using individual solutions of the arsenic species and few included As(III) and when they did, a first step in which As(III) was oxidized to As(V) was used before performing the adsorption as the As(V) species [31].

In this study, we have selected a commercially available MOF, Basolite^®^ F300, and a synthesized one, Nano-{Fe-BTC}, since both materials are formed with Fe (III) as the metal centre and trimesic acid (1,3,5-benzentricarboxylate, BTC) as the organic linker. The capacity of both iron-trimesate MOFs to adsorb inorganic arsenic species of As(III) and As(V) has been previously studied, showing that both the pH of the aqueous solution and the structural differences between these two MOFs affected the adsorption efficiencies [32]. The main objectives of this study were to characterize the kinetics and adsorption processes of organic arsenic species (MMA, DMA, ASA) by both Fe-BTC MOFs at different pHs, and to investigate the competition between them together with the inorganic species (arsenite and arsenate) towards both iron-trimesate MOFs in aqueous solutions. Additionally, the reusability of the adsorbents is evaluated by testing different eluent solutions.

## 2. Results and Discussion

Adsorption experiments were conducted to evaluate the removal efficiencies of both MOFs, Basolite^®^ F300 and Nano-{Fe-BTC}, for the different arsenic species. Preliminary tests were performed to determine the time needed to achieve equilibrium by monitoring the variation in the concentration of the arsenic species over time. All the studied compounds at the experimental conditions tested reached equilibrium in 30 min. However, the removal efficiencies of arsenobetaine, arsenocholine, and phenyl arsine oxide at all the studied pHs were lower than 10% and, therefore, no further study of these compounds was undertaken.

### 2.1. Adsorption Kinetics of Different Arsenicals

The kinetics of the adsorption of MMA, ASA, DMAA, As(V), and As(III) were studied in order to investigate the rate of the adsorption process and provide insights into the underlying sorption mechanism.

**Figure 1:**
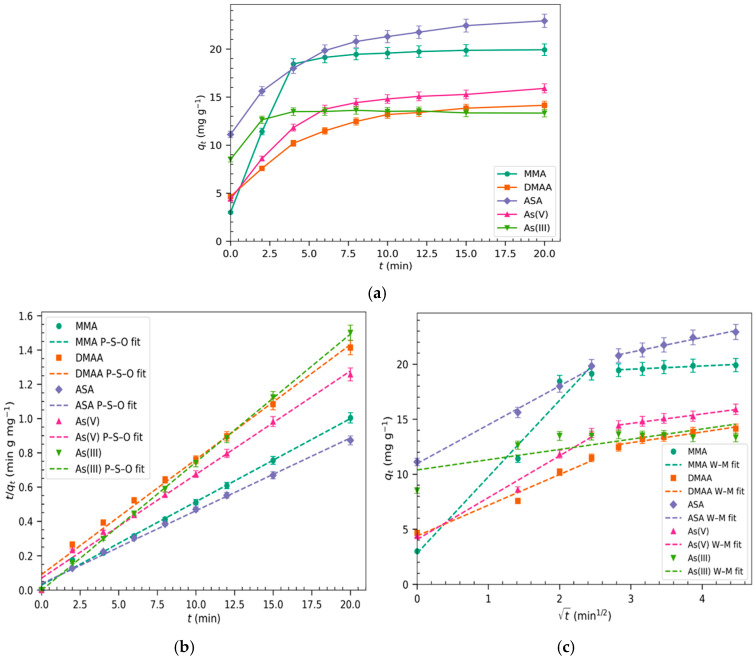
Kinetics of the adsorption processes: (**a**) effect of the contact time on the adsorption capacity (*q_t_*); (**b**) fit of the experimental data to the pseudo-second-order kinetic model; (**c**) application of the Weber–Morris kinetic model to the experimental data. Experimental conditions: C_0_ 10 mg L^−1^, 0.5 g L^−1^ Basolite^®^ F300; pH = 7 for MMA, DMAA, ASA, and As(V); pH = 11 for As(III); n = 3.

Adsorption of the arsenicals (MMA, ASA, DMAA, and As(V)) by Basolite^®^ F300 at pH 7 took place in under 12 min (Figure 1a), whereas in the case of As(III), saturation was reached in just 4 min at pH 11. To analyze the experimental kinetic data, adsorption kinetic models were then applied (Table 1). Firstly, pseudo-first-order (PFO) and pseudo-second-order (PSO) equations were used to obtain the kinetic constants, and, secondly, the Elovich and the Weber–Morris models were applied to elucidate the adsorption process (Figure 1b,c). The equations and results of the fitting of these models to the experimental data are summarized in Table 1. High correlation coefficients (R^2^ > 0.99) for all the arsenicals, which supported the goodness of fit, were obtained using the PSO model, suggesting, with the assumption that the MOF is homogenous, that the adsorption process is controlled by the surface availability. The rate at which the adsorption process approaches equilibrium, k_2_, gives insights into how quickly the adsorption processes occur, especially for MMA (k_2_ 0.082 g mg^−1^ min^−1^) as the k_2_ values for DMAA and As(V) are similar, 0.050 g mg^−1^ min^−1^ and 0.053 g mg^−1^ min^−1^, respectively. Even though As(III) adsorption takes place more rapidly, the rate constant k_2_ is the lowest, indicating that this MOF has less affinity to As(III) than the other arsenic species. The adsorbed maximum capacities *q_max_* (mg g^−1^) obtained with the PSO model are close to the experimental ones, suggesting that chemisorption is the dominant mechanism of the adsorption process. The application of the Elovich model to the kinetic adsorption data of MMA, DMAA, ASA, and As(V) shows two adsorption steps (Elovich M1 and M2), both with high R^2^ (Table 1). These findings are related to either the existence of two different interactions occurring between the adsorbent and adsorbate or the presence of two-step adsorption mechanisms.

In order to elucidate the diffusion process, we applied the Weber–Morris model. As can be seen in Figure 1c, two linear ranges can be distinguished for all the arsenic species, with the exception of As(III). The first one, which lasts until 4 min, is characterized by higher kWM1 values than the second and is associated with intra-particle diffusion of arsenic species following the order MMA > As(V) > ASA > DMAA. For the second step, at t > 5 min, the kinetic constants k_WM2_ are much lower and correspond to adsorption that takes place once the sorbent is enriched by arsenic species. The good fit of this model in both linear ranges (W-M M1 and M2) indicates the relevance of the diffusion of arsenic species into the pores of the MOF as a step that controls the adsorption process. However, the linear regression plot (W-M M1) did not pass through the origin (D_WM1_ ≠ 0), indicating that the adsorbate molecules diffused through a stagnant film or boundary layer surrounding the particles of Basolite^®^ F300. Thus, intra-particle diffusion is not the sole rate-limiting step of the adsorption [33]. Kinetic adsorption tests were only performed using Basolite^®^ F300, as in a recent study it was reported that the adsorption of As(III) at pH 11 and As(V) at pH 7 by Nano-{Fe-BTC} also followed a PSO model although the kinetic constant (k_2_) was ten times lower than for Basolite^®^ F300 given its lower porosity (Table S2) [32].

It is important to note that the adsorption of all the studied arsenic species, reaching equilibrium at just 30 min, is significantly faster than other similar studies in which these times have ranged from 3 to 48 h [20,25,29].

### 2.2. Adsorption Isotherms of Arsenic Species

**Figure 2:**
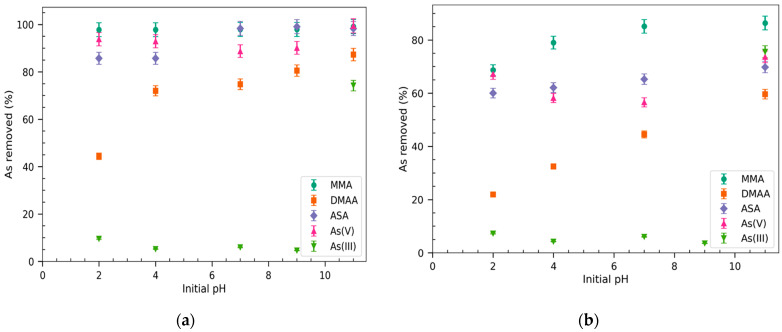
Adsorption isotherms and effect of initial aqueous solution pH the adsorption efficiency of Fe-trimesate MOFs for arsenical species: (**a**) Basolite^®^ F300; (**b**) Nano-{Fe-BTC}. Experimental conditions: C_0_ 10 mg L^−1^, 0.5 g L^−1^ adsorbent t = 30 min; n = 3.

Adsorption isotherm experiments were performed to evaluate the uptake capacity of Basolite^®^ F300 and Nano-{Fe-BTC} for the different arsenic species. The experiments were carried out at different initial pHs of 2, 4, 7, 9, and 11, and the results obtained are presented in Figure 2. Both Fe-BTC MOFs exhibited a similar uptake trend towards arsenic species with Basolite^®^ F300 being the more effective with removals of 100% for MMA and ASA. The difference between the removal efficiency of both MOFs is due to the greater surface area of Basolite^®^ F300 and its higher porosity (Table S2).

The characterization of the adsorption processes of arsenite and arsenate by both Basolite^®^ F300 and Nano-{Fe-BTC} was previously performed resulting in As(III) adsorption data that fits the Langmuir model, whereas As(V) adsorption data fits the Freundlich model better [32].

**Figure 3:**
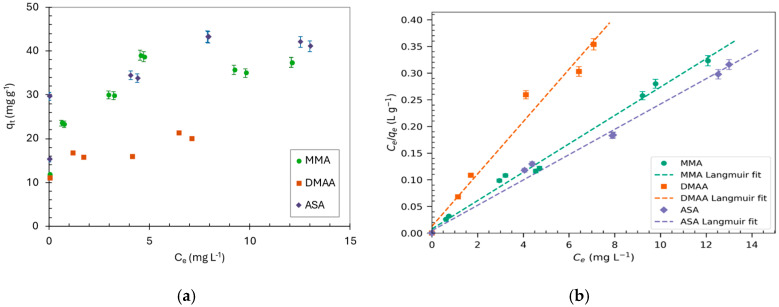
(**a**) Adsorption isotherms of organoarsenicals by Basolite^®^ F300; (**b**) fit of experimental adsorption data to Langmuir model. Experimental conditions: C_0_ 10 mg L^−1^, 0.5 g L^−1^ adsorbent t = 30 min, n = 3.

The adsorption isotherms for organoarsenical species were determined at pH 7 using Basolite^®^ F300 (Figure 3a). The experimental data was fitted using the Langmuir, Freundlich, Temkin, and Redlich–Peterson models (Table 2).

The Langmuir model provided a better fit (R^2^ > 0.9) than the Freundlich model, assuming a monolayer adsorption process where adsorbate molecules form a one-molecule-thick single layer on the adsorbent surface. These molecules are arranged throughout the homogeneous surface of the material at finite and energetically equivalent localized sites with no lateral interaction nor steric hindrance between the adsorbed molecules, even on adjacent sites. The *q_max_* required to form a complete monolayer is 37.6, 26, and 42.2 mg g^−1^ for MMA, DMAA, and ASA, respectively. The Redlich–Peterson model, which considers the possibility of a multilayer mechanism in addition to the monolayer (Langmuir), also gave a robust fit to the adsorption data. Since both the Langmuir and Redlich–Peterson models exhibit satisfactory fits, a thorough comparison using Akaike’s Information Criteria (AIC) was undertaken. The AIC analysis (Table 2) showed that the Langmuir model had a lower sum-of-squares (SS) for DMA (0.033) and ASA (0.001), compared to the Redlich–Peterson model, with 0.067 for DMA and 0.004 for ASA; therefore, the Langmuir model provides a superior fit to the adsorption data for these two organoarsenicals, whereas for MMA, the AIC showed a better fit with the Redlich–Peterson model. The goodness of fit with the experimental points can be visualized in Figure 3b. This suggests that the adsorption mechanism of organic arsenic species entails their attachment to the surface of Basolite^®^ F300, forming a monolayer, that in the case of MMA can evolve to a multilayer. The maximum sorption capacities of organoarsenicals by Basolite^®^ F300 at the experimental conditions (pH 7 and 0.5 g L^−1^ of adsorbent) follows the order ASA > MMA > DMAA.

Maximum adsorption capacities reported in other studies, which used different MOFs, some of which were functionalized and had varying metal centres and greater surface areas, are generally higher than those obtained in our study [6,29,33]. However, the use of Basolite^®^ F300 has the advantages of being commercially available and having very fast kinetics in comparison with synthesized MOFs. Moreover, the synthesis of Basolite-like MOFs such as Nano-{Fe-BTC} is simple and can be carried out by a green procedure [34]. Furthermore, it should be noted that comparison of adsorption capacities is complicated by the fact that the obtained values are highly dependent on multiple factors including the adsorbent/adsorbate ratio, and the experimental conditions, such as the pH of the aqueous media and the presence of competing species, the adsorbate concentrations, the speed of agitation, the amount of adsorbent, and the physico-chemical characteristics of both the adsorbent and the adsorbate.

### 2.3. The Effect of pH

The pH can significantly influence adsorption behaviour as it determines the metal speciation as well as the physico-chemical properties of the surface of the adsorbent material.

As can be seen in Figure 2, the removal percentages of Basolite^®^ F300 towards arsenic species depends on the pH of the aqueous solution for ASA, DMAA, and As(III), whereas for MMA and As(V), no significant differences were observed in the whole pH range. However, in the case of Nano-{Fe-BTC}, the removal efficiency for all the arsenic species showed a pH dependence more noticeable for DMAA and As(III). It is important to remark that the pH values of the x-axis in Figure 2 correspond to the initial pH of the arsenic aqueous solutions. The acidity of Fe-BTC MOFs, which is related to the acid–base properties of 1,3,5-benzene tricarboxylate (BTC) ligands, resulted in a decrease of two pH units when the sorbent concentration was of 0.5 g L^−1^ at initial pH = 11. The pH at the point zero charge of the Fe-BTC MOFs was determined to be 3.8 for Basolite^®^ F300 and 4 for Nano-{Fe-BTC} [32]. The stability of the Fe-BTC MOFs at the experimental conditions before and after arsenic adsorption was evaluated by recording PXRD spectra showing no affectation of the crystalline nature of MOFs [32]. The thermal stability of both Fe-BTC MOFs, which is an indicator of structural stability, was assessed by thermogravimetric analyses (TGA) and stability tests in water were also performed [34].

The adsorption behaviour of As(III) is similar for both Fe-BTC MOFs as at initial pHs between 2 and 9. As(III) uptake is insignificant in this pH range due to the predominance of neutral species, H_3_AsO_3_, in the aqueous media (Figure S2). The adsorption percentage of As(III) increases substantially until 75% when the pH of the aqueous solution was adjusted to 11 given that the anionic species H_2_AsO_3_^−^ predominates at pHs > 9.5 (Figure S2). The adsorption of arsenite on both Basolite^®^ F300 and the synthesized Nano-{Fe-BTC} is likely due to the electrostatic interaction of the soft Lewis base H_2_AsO_3_^−^ and the centred iron cations of the MOFs. In the case of As(V), the high removal percentages over the entire pH are explained by the coordination between arsenate and the incompletely coordinated cationic Fe (Lewis acid) in the cluster through the formation of Fe−O−As bonds, as has been reported in several studies and characterized by FTIR and XPS analyses [6,32,34]. Additionally, electrostatic interactions between the positive charge of the centred Fe^3+^ and either H_2_AsO_4_^−^ at pH 2.5-8 or HAsO_4_^2−^ at pHs from 8 to 11 (Figure S2) contribute to the arsenate uptake for both MOFs. The trend of As(V) uptake by Nano-{Fe-BTC} showed its maximum adsorption capacity for As(V) at pH 2 and further increases in the pH value led to a slight reduction in efficiency at pH 4 and an increase at pH 11. This fact can be explained by the lesser porosity and surface area of Nano-{Fe-BTC} MOF (Table S2) that is negatively charged at pH > 4 due to the deprotonation of the trimesic acid linkers favouring repulsion forces with negatively charged species such as H_2_AsO_4_^−^ and HAsO_4_^2−^ [32].

The uptake of MMA, DMAA, and ASA by both Fe-BTC MOFs is mainly attributed to the coordination between Fe(III) and the arsenic species through the formation of Fe-O-As bonds. It has been reported that the number of coordination sites in arsenic species depends on the number of hydroxyl groups in their molecular structures and, hence, the formation of monodentate mononuclear and bidentate binuclear complexes between organoarsenical species with the Fe-MOFs depends on the number of hydroxyl groups. ASA and MMA have two hydroxyl groups—which can offer three possible edge sites to form monodentate mononuclear complexes—while DMAA has one hydroxyl group and two possible sites to form monodentate mononuclear complexes and one site to form bidentate complexes [6]. The fact that removal percentages of AsB, AsC, and PhAs by Fe-BTC-MOFs were lower than 10% supports the relationship between effective arsenic adsorption and the presence of hydroxyl groups in the adsorbate molecules. The maximum sorption capacities of Basolite^®^ F300, calculated with the Langmuir model, follows the order ASA > MMA > DMAA (Table 2) and can be associated with the number of hydroxyl groups. The higher adsorption capacity of Basolite^®^ F300 for ASA than MMA is explained by π-π stacking interactions between the aromatic ring of ASA and aromatic moieties of 1,3,5-benzene tricarboxylate (BTC) ligands of the Fe-BTC MOFs, which was described for the sorption of ASA by MIL-101(Fe) and demonstrated by XPS spectra [6]. Additionally, FTIR spectra demonstrated the interaction of arsenic species with the iron centres of the Fe-BTC MOFs through the appearance of a band at 825 cm^−1^, attributed to Fe–O–As [32]. The similarity of the IR spectra before and after the uptake of arsenic species at initial pHs of 7 and 11 also demonstrate that both Fe-BTC MOF structures are stable at these experimental conditions.

The relevance of the Fe(III) metallic centre of the MOFs for an efficient uptake of arsenic species was demonstrated when three analogue MOFs with similar porosities, MIL-100-Cr, MIL-100Al, and MIL-100Fe, were evaluated for ASA and ROX sorption, obtaining significant adsorption capacities only in the case of MIL100(Fe) [27]. MIL100-Fe and Basolite^®^ F300 have similar structures, and both have CUSs that allow the uptake of arsenate [23,32,35].

The studied arsenic compounds have acid–base characteristics (Table S1) that can affect their sorption behaviour. As can be seen in Figure 2, the uptake of MMA by the two Fe-MOFs is the most affected by the pH of the aqueous solution as it increases when the pH increases, whereas the increase is lesser for ASA and DMAA sorption by Nano-{Fe-BTC}. The deprotonation of DMAA at pHs > 6 and ASA at pHs > 4 (see pKas in Table S1), which resulted in the formation of negatively charged species that can electrostatically interact with Fe^3+^, could explain the increase in uptake of these arsenic species.

### 2.4. Competition of the Arsenic Species for the Adsorbent

**Figure 4:**
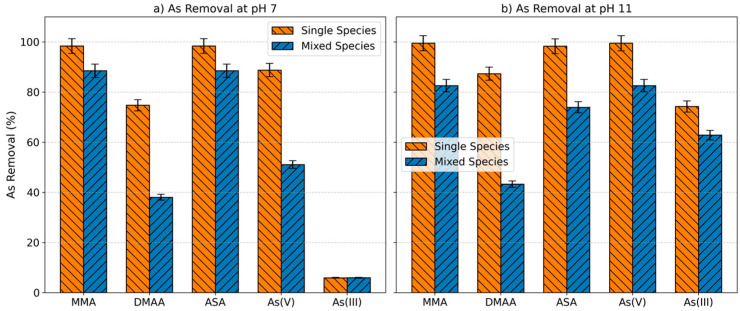
Comparison of removal percentage between single and multi-arsenical species solutions at initial pH = 7 and initial pH = 11. Experimental conditions: C_0_ 10 mg L^−1^. Basolite^®^ F300 0.5 g L^−1^; n = 3.

Adsorption tests with multi-component solutions containing all the arsenic species were also conducted and the removal percentages were then compared with those obtained for each individual species at the same experimental conditions (10 mg L^−1^ and 0.5 g L^−1^ of adsorbent) at initial pHs 7 and 11 (Figure 4). There is a decrease in arsenic uptake in the mixed solutions for all the arsenic species at the two pHs, except for As(III) at pH 7. However, this decrease is less than 10% for MMA and ASA species at pH 7 and As(III) at pH 11 and higher than 30% for DMAA at both pHs and As(V) at pH 7. These results confirm the competition of arsenic species to coordinate with the Fe^3+^ centres in the Fe-BTC MOF cluster.

### 2.5. Competing Ions in the Adsorption of Arsenic Species by Fe-BTC MOFs

The presence of other ions with arsenic species in the same solution can affect the efficiency of the adsorbent and its potential practical applicability. Anions such as Cl^−^, F^−^, NO_3_^−^, SO_4_^2−^, CO_3_^2−^, SiO_3_^2−^, and PO_4_^3−^, which can be found in waters, may compete with arsenic species during the adsorption process. Of these, only PO_4_^3−^ has been reported as significantly affecting the adsorption capacity of Fe-MOFs to adsorb As(V), although CO_3_^2−^ and SiO_3_^2−^ slightly affected the adsorption of ASA and DMAA. These results are explained by the formation of inner-sphere complexes between arsenic species and the adsorbent [6,36]. Therefore, the effect of PO_4_^3−^ on As(III) and As(V) removal was investigated by varying the initial concentration of PO4^3−^ while the concentration of inorganic arsenic species was maintained at 10 mg L^−1^. The presence of phosphate (10 mg L^−1^) reduced the removal percentage of As(III) and As(V) by 18.5% and 6%, respectively, and the increase of PO_4_^3−^ concentrations to 100 and 300 mg L^−1^ led to a 20% decrease in the removal percentage for As(V) and a 10% decrease for As (III). The competition between arsenite, arsenate, and PO_4_^3−^ presumably results from their chemical similarities as P and As have similar electronegativity and arsenate and phosphate are able to form inner-sphere complexes with the Fe-MOFs [6].

**Figure 5:**
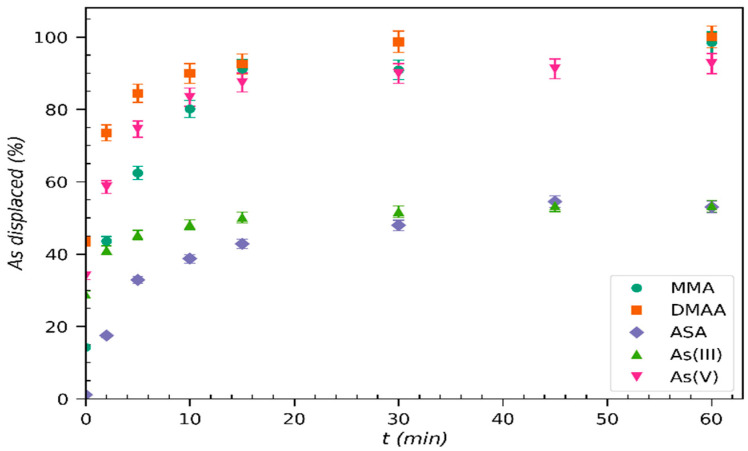
Desorption of arsenical species from Basolite^®^ F300 after adding 50 mL of 100 mg L^−1^ Na_2_HPO_4_ to 50 mL of solution of all arsenical species in equilibrium with loaded adsorbent, n = 2.

Desorption of the individual arsenic species from Basolite^®^ F300 was studied by adding a Na_2_HPO_4_ solution as described in the methodology (Figure 5). It can be seen that quantitative elutions for As(V), DMAA, and MMA were obtained after 60 min, whereas for ASA and As(III), elution was around 50%. However, the brown-orange colour of the solution indicates that the adsorbent is partially decomposed.

### 2.6. Elution with Methanol: Desorption and Reuse

**Figure 6:**
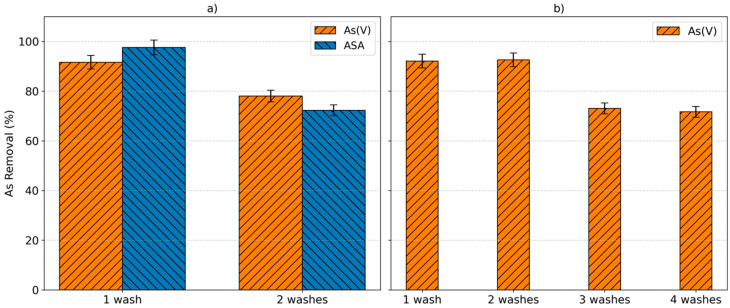
The reusability of Basolite^®^ F300 after performing the adsorption of selected arsenical species: (**a**) the removal percentages of As(V) and ASA after washing the loaded adsorbent with methanol (1 wash) and after reusing the adsorbent twice (2 washes); (**b**) the removal percentages of As(V) uptake after washing the sorbent with acidic methanol (0.1% formic acid) and reusing the adsorbent for four cycles. Experimental conditions for adsorption: C_0_ 10 mg L^−1^ at pH 7 and 0.5 g L^−1^ for the first adsorption cycle; n = 2.

Investigation into the reuse of adsorbents is important in order to promote resource conservation, cost reduction, and sustainability and reduce other environmental impacts. Few studies have reported the reusability of Fe-MOFs for arsenic species. Li et al. found that 80% of the adsorbed As(V) could be desorbed from MIL-101-Fe by NaOH elution and that only 60% of adsorption efficiency was maintained after eluting three times [6]. Jun et al. reported that MIL-100(Fe) can be used three times for the adsorption of ASA after washing the used material with acidic ethanol given that the coordinated organoarsenical species are replaced by water through hydrolysis aided by the proton [27]. In this study, the reusability of Basolite^®^ F300 was evaluated using methanol. As can be seen in Figure 6, the efficiency of this MOF in absorbing As(V) and ASA is reduced by 14% and 25%, respectively, solely as a result of washing with methanol. To ameliorate this loss of efficiency, more experiments were conducted in which methanol was acidified with 0.1% of formic acid (Figure 6b). The performance of recycled Basolite^®^ F300 improved towards As(V) and reached the maximum adsorption capacity after one wash, proving that protons play an important role in the exchange of arsenate by water (ligand exchange). After performing two cycles, the adsorption percentage of As(V) decreased from 95% to 75%, maintaining this percentage for two more cycles. Furthermore, there is no deterioration observed when this Fe-MOF is reused four times as the sorbent maintained its adsorptive properties and the solution remained uncolored, showing that iron was not released from the sorbent. The efficiency of methanol to elute dyes and pharmaceuticals adsorbed in Fe-BTC MOFs was demonstrated in previous studies [34,37].

## 3. Materials and Methods

### 3.1. Chemicals

The arsenic compounds monosodium methylarsonate (MMA, Sigma-Aldrich PS-429, Darmstadt, Germany), cacodylic acid (DMAA, Acros 214970100, Fisher Scientific GmbH, Schwerte, Germany), and sodium 4-Aminophenylarsonic acid (ASA, TCI A053, TCI Deutschland GmbH, Eschborn, Germany) and inorganic species As_2_O_5_ (Acros 366310250, Fisher Scientific GmbH, Schwerte, Germany) and As_2_O_3_ (Sigma-Aldrich A1010, Darmstadt, Germany), all of which were HPLC grade, were purchased and used. Arsenobetaine bromide (AsB, LGC/TRCA778500, Burglinton, ON, Canada), arsenocholine bromide (AsC, LGC/TRC-A778550, Burglinton, ON, Canada), and phenyl arsine oxide (PhAs, Sigma-Aldrich P3075, Darmstadt, Germany) were also tested. The structures and physical–chemical proprieties of all these compounds are presented in Table S1 (Supplementary Materials). Basolite^®^ F300 was purchased from Sigma-Aldrich (Chemie Gmbh, Steinheim, Germany) and Nano-{Fe-BTC} was synthesized as has been previously described following green chemistry rules [34]. HPLC grade (≥99.8%) was obtained from Honeywell (Seelze, Germany), ammonia, ammonium carbonate, and formic acid were from VWR International (Leuven, Belgium).

The specific surface areas, pore size distribution, and pore volumes of Basolite^®^ F300 and Nano-{Fe-BTC} were measured by the Brunauer, Emmett, and Teller method (BET) at 77K in the range of 0.02 ≤ *P*/*P*_0_ ≤ 1.00 on a Gemini V 2365 Model from Micrometrics International Corporation (Norcross, GA, USA). The morphology and size of the synthesized materials were examined using transmission electron microscopy (TEM) (JEOL 2010, Tokyo, Japan). A scanning electron microscope (JEOL JSM 6300, Tokyo, Japan) with a resolution of 3.5 nm combined with an energy-dispersive X-ray spectrometer (Oxford 6699ATW, Oxford, Oxfordshire, UK) was used to determine the surface composition of the materials [32]. FTIR analyses of both sorbents were carried out in the 600–3600 cm^−1^ region using an Agilent Cary 630 FTIR spectrometer (Agilent, Santa Clara, CA, USA) equipped with a diamond attenuated total reflectance (ATR) accessory. The main characteristics of both Fe-based MOFs such as porosity, surface area, and pore volume are collected in Table S2 and Figure S1 of the Supplementary Materials.

Arsenic species stock solutions of 100 mg L^−1^ were prepared by dissolving MMA, DMAA, ASA, AsB, AsC, and As_2_O_5_ in Milli-Q water. As(III) stock solution was obtained by dissolving As_2_O_3_ in a 5 M NaOH solution. Phenyl arsine oxide stock solution was prepared by adding 5 drops of DMSO (dimethyl sulfoxide, Sigma-Aldrich, Darmstadt, Germany) to a certain amount of solid and then diluted with Milli-Q water. The resulting solution was subsequently heated at 60 °C for 10 min to ensure complete dissolution. The working standard solutions were prepared by diluting the stock solutions. HNO_3_ (0.1 M) and NaOH (0.1 M) were used to adjust the pH of the working solutions. All experiments were conducted using ultrapure water from a Milli-Q-system (Merck-Millipore, Billerica, MA, USA) and used directly without purification.

The pH of the solutions was measured using a Metrohm pH metre 844 with a pH electrode (Mettler-Toledo GmbH, Giessen, Germany) which was previously calibrated with buffer solutions of pH 4, pH 7, and pH 10.

### 3.2. Batch Adsorption Experiments

Batch arsenic species studies were performed under controlled conditions at a room temperature of 25 °C. In total, 10 mg of each sorbent (Basolite^®^ F300 and Nano-{FeBTC}) was individually added to 20 mL of 10 mg L^−1^ arsenic solutions, comprising MMA, DMAA, ASA, AsB, AsC, PhAs, As(III), or As(V) solutions and stirred using an RCT magnetic stirrer (IKA, RCT basic, IKA-Werke GmbH, Staufen, Germany) until the desired contact time was reached. The adsorption performance was studied in the pH range of 2 to 11.

For the kinetic studies, 50 mg of each sorbent was mixed with 100 mL of each arsenic solution at pH 7 while being stirred, except at pH 11 for As(III). At different contact times ranging from 0 to 20 min, a 0.5 mL volume of the solution was withdrawn and analyzed.

In order to study single component sorption isotherms, the initial arsenic concentration effect was investigated. In total, 10 mg of adsorbent was dispersed in 20 mL of different arsenic solutions with concentrations ranging from 5 to 25 mg L^−1^, stirring until equilibrium was reached.

Adsorption experiments using multiple components solutions were performed to study the competition towards the adsorbent of the different arsenic species. A solution containing 10 mg L^−1^ of each arsenic compound MMA, DMA, ASA, As(III), and As(V), with pH adjusted to the target value (pH 7 or 11), was prepared. Then, 10 mg of Basolite^®^ F300 was added to 20 mL of the multi-component solution, and the mixture was agitated for 2 h.

The arsenic uptake rate in each experiment and the amount of adsorbed arsenic at the equilibrium q_e_ (mg g^−1^) were calculated according to Equation (1) and Equation (2), respectively.

(1) $$Adsorption\;\left(\%\right)=\frac{\left(C_{i}-C_{eq}\right)}{C_{i}}\cdot 100$$

where C_i_ and C_eq_ (mg L^−1^) are the concentrations of arsenic at the initial and equilibrium times, respectively.

(2) $$q_{e}=\frac{\left(\left(C_{i}-C_{eq}\right)\cdot V\right)}{W}$$

where V (L) is the volume of the solution, and W (g) is the mass of dry adsorbent used.

### 3.3. Desorption and Reusability Experiments

Sorption of the different arsenic species was first performed using 50 mL of 10 mg L^−1^ of individual arsenic solutions at pH 7, except for As(III) at pH 11, in contact with 0.5 g L^−1^ of Basolite^®^ F300 and stirred for 30 min until equilibrium was achieved. In order to study the desorption from the adsorbent, 50 mL of a 100 mg L^−1^ Na_2_HPO_4_ (Sigma-Aldrich, Darmstadt, Germany) solution was then added and stirred for 60 min. At prefixed times, 0.5 mL of this mixture was collected and analyzed to determine the concentrations of the eluted arsenic compounds.

The reusability of Basolite^®^ F300 was investigated by washing the adsorbent with methanol or acidified methanol. In the first case, a solution containing 10 mg L^−1^ of As(V) and 10 mg L^−1^ of ASA at pH 7 was used to perform the sorption process with 0.5 g L^−1^ of Basolite^®^ F300. After equilibrium was reached, the loaded sorbent was filtrated, dried, and washed with methanol after ensuring that both arsenic species had been eluted from the adsorbent. It was reused twice in the same conditions. The same procedure was followed to evaluate the reusability of Basolite^®^ F300 after sorption of 10 mg L^−1^ of As(V) with 0.5 g L^−1^ of Basolite^®^ F300 and washing the adsorbent with acidified methanol with 0.1% formic acid. In this case, four cycles were performed.

### 3.4. Chromatographic Analyses

Arsenic species were separated and determined in all extracts by an HPLC single quadrupole MS system (Agilent 1100 Series LC/MSD, Agilent, Santa Clara, CA, USA) equipped with an anion exchange column Metrosep A Supp 5–150/4.0 (150 × 4.0 mm, 5 µm) (Metrohm, Herisau, Switzerland). Separation was performed under isocratic conditions using a pre-mixed eluent (90:10% *v/v* (water/acetonitrile) and 10 mM (NH_4_)_2_CO_3_ (adjusted at pH 10 using 25% NH_3_) at a flow rate of 0.2 mL/min and a column temperature of 45 °C. The standard injection volume was 5 µL. The sample was previously filtered with 0.45 µm Ø 25mm LLG-syringe Spheros filters (LLG Labware, Bad Salzuflen, Germany).

Arsenic species were identified using the SIM mode: MMA, DMAA, ASA, AsB, AsC, and PhAs as [M+H]^+^ and As(III) and As(V) as [M−H]^−^. Ionization was performed in API-ES mode with the following source settings: capillary voltage 3500 V, gas flow 12.5 mL/min, nebulizer pressure 55 psi, source temperature 350 °C, fragmentor voltage 57 V (positive polarity), and 70 V (negative polarity). Chromatograms were recorded at 220 nm.

Agilent Chemstation B.04.03 (Agilent, Santa Clara, CA, USA) was used in the treatment of the chromatographic data. To enhance the resolution of the peaks, chromatograms were refined using the functionalities within the Agilent ChemStation software.

Calibration curves were plotted by analyzing six individual arsenic standard solutions with final concentrations ranging from 0.5 to 30 mg L^−1^. These standard calibration solutions were prepared at the same pH and matrix media of the experimental solutions by dilution with Milli-Q water of 100 mg L^−1^ standard arsenic solutions. The retention times, detection mode, calibration curve equations, and limits of quantification (LOQ), estimated at a signal-to-noise (S/N) ratio ≥ 10, for each arsenic compound are presented in Table S3.

## 4. Conclusions

This study demonstrated the efficiency of two Fe-trimesate MOFs, Basolite^®^ F300 and Nano-{Fe-BTC}, in adsorbing organoarsenicals (MMA, DMAA, and ASA) and inorganic arsenic species (arsenite and arsenate) from water across a broad range of pHs. However, the sorption percentages obtained with Basolite^®^ F300 were higher than those obtained with Nano-{Fe-BTC} at all the studied pHs, reaching removal percentages > 90% for MMA, ASA, and As(V). These results are explained by the greater surface area and the higher porosity of Basolite^®^ F300. Arsenic species uptake by Nano-{Fe-BTC} was more affected by the pH of the aqueous solution than Basolite^®^ F300. However, both Fe-BTC MOFs displayed the same behaviour in adsorbing As(III). Moreover, kinetic and isotherm studies reveal that Basolite^®^ F300 rapidly adsorbs all these arsenic species, demonstrating excellent adsorption rates and capacities, and exhibiting a chemisorption-dominated process. The maximum sorption capacities of organoarsenicals by Basolite^®^ F300 at the experimental conditions follows the order ASA > MMA > DMAA, which correlates with the number of hydroxyl groups of these organic compounds. This finding supports the assumption that the main adsorption mechanism of organoarsenicals by Fe-BTC MOFs is the coordination through the formation of Fe−O−As bonds, demonstrated in the case of inorganic arsenic species [32]; π-π interactions in the case of ASA can also contribute to the arsenic uptake.

Basolite^®^ F300 displayed the ability to simultaneously remove all studied arsenic compounds, although the competition between them resulted in lower removal rates compared with those of the individual arsenic species. However, the decrease in the removal rate was less than 10% for MMA and ASA at pH 7.

The ease with which this adsorbent can be recycled with an acidic methanol eluant adds to its versatility. The combination of efficient adsorption, fast kinetics, stability, and recyclability makes Fe-BTC MOFs a promising and practical adsorbent for the uptake of organic and inorganic arsenic species.

In conclusion, in providing valuable insights into the challenges faced in reducing contamination by arsenic, the significance of the potential of Fe-BTC MOFs such as commercially available Basolite^®^ F300 and synthesized Nano-{Fe-BTC} to adsorb all arsenic species can be seen.

## Figures and Tables

**Table 1:** Kinetic adsorption study of MMA, DMAA, ASA, and As(V) at pH 7, and As(III) at pH 11 onto Basolite^®^ F300 MOF.

Model	Equation		MMA	DMAA	ASA	As(V)	As(III)
**PFO**	$\log\left(q_{e}-q_{t}\right)=\log q_{e}-\frac{K_{1}}{2.303}t$	*q_e_* (mg g^−1^)	6.95	9.29	10.11	8.70	0.672
		K_1_ (min^−1^)	0.223	0.080	0.122	0.138	0.092
		R^2^	0.841	0.895	0.968	0.934	0.198
**PSO**	$\frac{t}{q_{t}}=-\frac{1}{K_{2}{\;q}_{e}^{2}}+\frac{1}{q_{e}}\;t$	*q_e_* (mg g^−1^)	20.6	14.9	23.53	16.6	13.4
		k_2_ (g mg^−1^ min^−1^)	0.082	0.050	4.94 × 10 ^−4^	0.053	−2.93
		R^2^	**0.996**	**0.992**	**0.995**	**0.994**	**0.999**
**Elovich**	$q_{t}=\frac{1}{\beta }Ln\left(\alpha \beta \right)+\frac{1}{\beta }\ln\left(t\right)$	α (mg g^−1^ min^−1^)	32.8	30.7	125.32	29.3	1.07 × 10^4^
**M1**							
		β (g mg^−1^)	0.047	0.114	0.092	0.084	0.339
		R^2^	**0.961**	**0.998**	**0.98**	**0.995**	**0.583**
**Elovich**		α′ (mg g^−1^ min^−1^)	7.97 × 10^6^	612 × 10^3^	3734.79	4.98 × 10^4^	
**M2**							
		β′ (g mg^−1^)	0.807	0.244	0.179	0.280	
		R^2^	**0.955**	**0.954**	**0.993**	**0.983**	
**W-M**	$q_{t}=K_{WM}{\;t}^{0.5}+D_{WM}$	K_WM1_ (mg g^−1^ min^−1/2^)	6.95	2.79	3.53	3.80	0.932
**M1**							
		D_WM1_	2.810	4.379	10.96	4.085	10.41
		R^2^	**0.970**	**0.972**	**0.996**	**0.981**	0.592
**W-M**		K_WM2_ (mg g^−1^ min^−1/2^)	0.295	0.975	1.338	0.862	
**M2**							
		D_WM2_	18.66	9.934	17.07	12.04	
		R^2^	**0.926**	**0.924**	**0.982**	**0.987**	

**Table 2:** Models and their parameters for organoarsenical species adsorption onto Basolite^®^ F300.

Model	Equation		MMA	DMAA	ASA
Langmuir	$\frac{C_{e}}{q_{e}}=\frac{1}{q_{max}k_{l}}+\frac{1}{q_{max}}C_{e}$	*q_max_* (mg g^−1^)	37.6	25.9	42.2
		K_L_ (L mg^−1^)	3.50	4.69	5.15
		R^2^	**0.994**	**0.966**	**0.993**
		SS	**0.001**	**0.033**	**0.001**
Freundlich	qe=KfCe1n	*n*	6.1	5.7	3.4
		*K_F_* (L g^−1^)	25.7	21.1	21.5
		R^2^	0.811	0.389	0.663
Temkin	$q_{e}=BLn\left(A\right)+B\ln\left(C\right)$	A (L mg^−1^) B (J mol^−1^)	7.11 × 10^−25^ 0.069	7.21 × 10^−10^ 0.032	3.87 × 10^−20^ 0.040
		R^2^	0.777	0.357	0.761
Redlich–Peterson	$\log\left(\frac{C_{e}}{q_{e}}\right)=\log k_{R}+\beta_{R}TLn\left(C_{e}\right)$	β_RP_ K_RP_ (g L^−1^)	0.033 0.039	0.033 0.047	0.033 0.037
		R^2^	**0.991**	**0.935**	**0.975**
		SS	0.012	0.067	0.004

SS: Squares sum of errors.

## Data Availability

The raw data supporting the conclusions of this article will be made available by the authors on request.

## Supplementary Materials

The following supporting information can be downloaded at: https://www.mdpi.com/article/10.3390/molecules30214198/s1. Table S1: Chemical structures and physico-chemical characteristics of arsenical compounds investigated. Table S2: The pore parameters of Basolite^®^ F300 and Nano-{Fe-BTC}. Table S3: (A) Summary of analytical parameters of HPLC-MS method for determination of arsenical species and (B) retention times and detection mode by MS. Figure S1: Characterization of Basolite^®^ F300 and Nano-{Fe-BTC}. (a) TEM images at 20 nm, (b) FTIR spectra, and (c) porosimetry. Figure S2: Speciation diagrams of As(III) and As(V).
