# Supplementary material for: Iron-Based Metal–Organic Frameworks for the Removal of Different Organic and Inorganic Arsenic Species from Water: Kinetic and Adsorption Studies
Source: Molecules. 2025 Oct 27;30(21):4198. doi: 10.3390/molecules30214198 (PMC12608843; doi:10.3390/molecules30214198)
Supplement: Supplementary file 1 [file molecules-30-04198-s001.zip › molecules-3833998-supplementary.pdf]

Table S1. Chemical structures and physico-chemical characteristics of the arsenical compounds investigated.

Compound Name	Chemical Structure	Oxidation State	pKa(s)	Solubility (in water)
Disodium methyl arsonate (MMA)	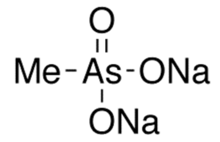	+5	pka_1=4.1<br>pka_2=8.94	300 g/l
Cacodylic acid (DMAA)	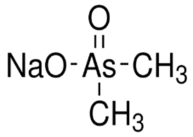	+5	pKa = 6.2	200 g/L
Sodium 4-Aminophenylarsonate (ASA)	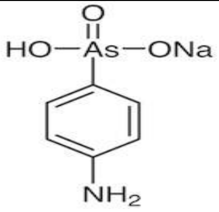	+5	pK_1=4.02-4.36<br>pka_2=8.7	1 g/L
Arsenic(III) Arsenite	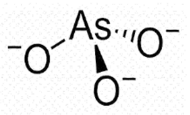	+3	pka_1=9.23<br>pka_2=12.13<br>pka_3=13.4	1.2-3.7 mg/L
Arsenic(V) Arsenate	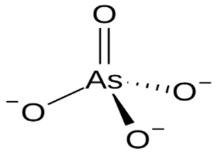	+5	pka_1=2.19<br>pka_2=6.98<br>pka_3=11.53	65.8 g/100 mL
Arsenobetaine	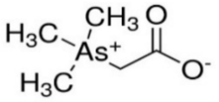	+5	pKa =2.18	soluble
Arsenocholine	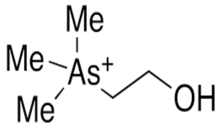	+5	-	soluble
Phenylarsine oxide	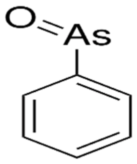	+3	-	Non-soluble

Table S2: The pore parameters of Basolite® F300 and Nano-{Fe-BTC}

Parameters	Nano-{Fe-BTC}	Basolite® F300
Surface Area (m^2/g)	431.6	840
Micropore Area (m^2/g)	251.7	235.9
External Surface Area (m^2/g)	175.4	130.9
Micropore volume (cm^3/g)	0.115	0.107
Pore Volume (cm^3/g)	0.527	0.206
Pore Size (Å)	49.3	22.4
Porosity %	10.12	29.95

Surface area obtained by BET equation at P/Po range from 0.005 to 0.05; Micropore area and Micropore volume and external surface area calculated by t-plot method; Pore volume calculated based on single-point N_2 adsorption at P/Po: ~0.99.

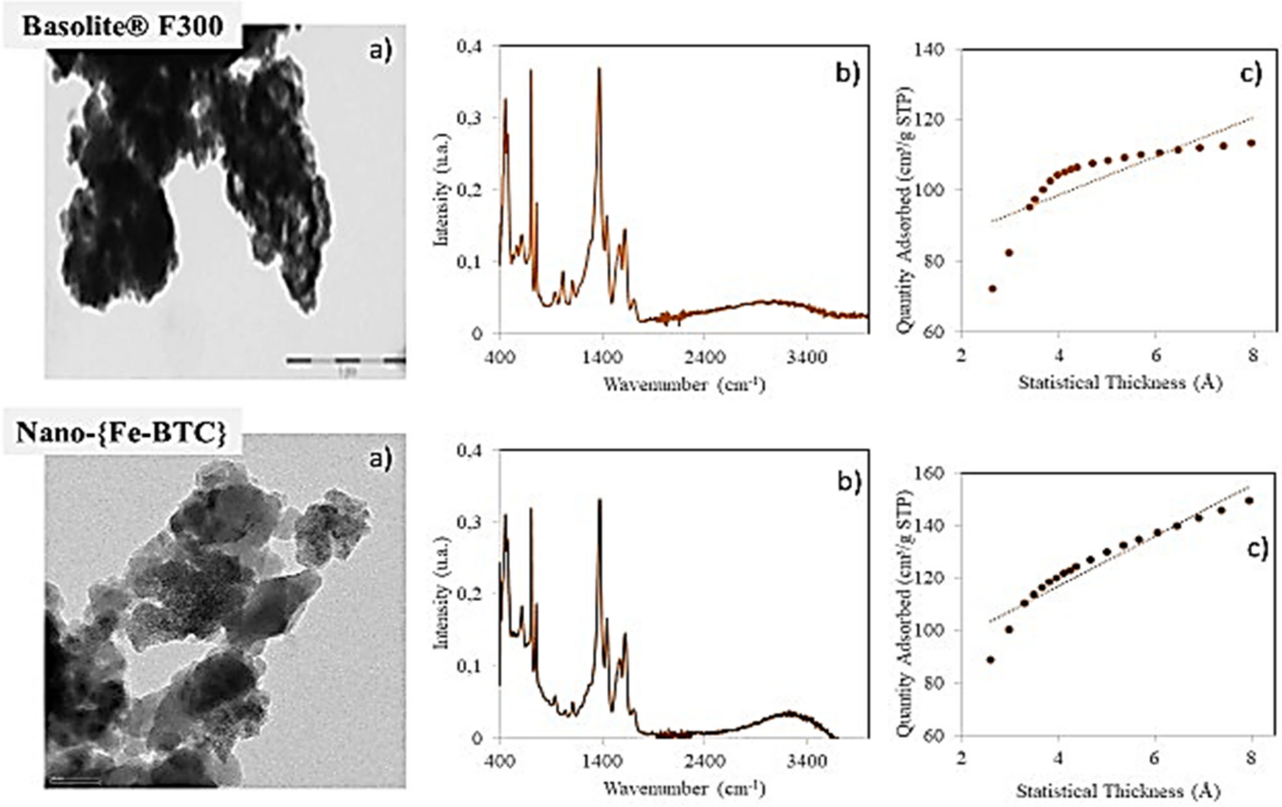

Figure S1. Characterization of Basolite® F300 and Nano-{Fe-BTC} **a)** TEM images at 20 nm, b) FTIR spectra, and c) porosimetry

Table S3. A) Summary of analytical parameters of HPLC-MS method for the determination of arsenical species and B) retention times and detection mode by MS.

A)

**Analyte**	**a (mg)**	s_a	**b (L/mg)**	s_b	**LOD (mg/L)**	**LOQ (mg/L)**	**CV (%)**	R^2
MMA	13186	397.8	39626	78.0	0.03	0.10	1.16	0.9999
DMA	1099379	114520	520767	22240	0.73	2.20	3.40	0.9982
ASA	16432	6449	164178	1265	0.13	0.39	3.14	0.9999
As(III)	-919,5	167.5	6716.2	32.4	0.08	0.25	2.59	0.9989
As(V)	-2277	1826	53751	335.7	0.11	0.34	2.28	0.9999

B)

**Compound Name**	**Retention time (t_r) min**	**Detection Mode (MS)**
MMA	9.2	Positive mode (141 m/z)
Cacodylic acid (DMAA)	3.1	Positive mode (139 m/z)
ASA	11.1	Positive mode (218 m/z)
Arsenic(III)	3.7	Negative mode (125 m/z)
Arsenic(V)	14	Negative mode (141 m/z)
Arsenobetaine	2	Positive mode
Arsenocholine	1.9	Positive mode
Phenylarsine oxide	5.7	Positive mode

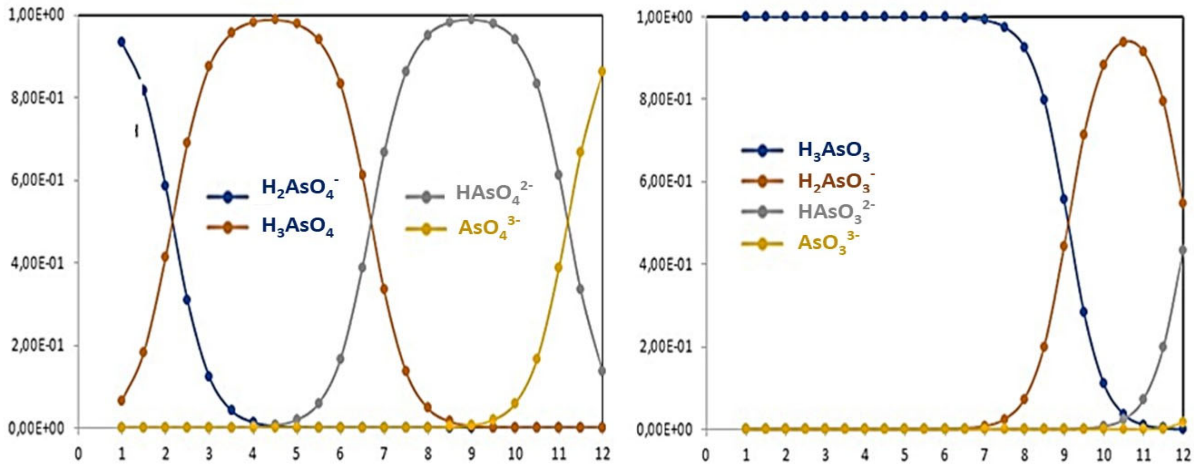

Figure S2. Speciation diagrams of As(III) and As(V).
